# Pathogenesis of tubercular retinal vasculitis: An ongoing quest

**DOI:** 10.1016/j.jctube.2026.100583

**Published:** 2026-01-14

**Authors:** Ikhwanuliman Putera, Rina La Distia Nora, Saskia M. Rombach, P. Martin van Hagen, Willem A. Dik

**Affiliations:** a Department of Ophthalmology, Faculty of Medicine, Universitas Indonesia – Cipto Mangunkusumo Hospital, Jakarta, Indonesia; b Laboratory of Medical Immunology, Department of Immunology, Erasmus University Medical Centre, Rotterdam, the Netherlands; c Department of Ophthalmology, Erasmus University Medical Centre, Rotterdam, the Netherlands; d Department of Internal Medicine, Section Allergy & Clinical Immunology, Erasmus University Medical Centre, Rotterdam, the Netherlands; e Department of Immunology, Faculty of Medicine, Chulalongkorn University, Bangkok, Thailand; f Department of Internal Medicine, Faculty of Medicine, Universitas Indonesia, Jakarta, Indonesia; g Reinier Haga Medisch Diagnostisch Centrum (RHMDC), Laboratory Medical Immunology, Delft, the Netherlands; h University of Indonesia Academic Hospital (RSUI), Depok, West Java, Indonesia

Retinal vasculitis is a well-recognized clinical feature that is consistently included in the diagnostic criteria for ocular tuberculosis (OTB), both in the original Gupta et al. classification [1] and the more recent criteria by the Standardized Uveitis Nomenclature (SUN) working group [2]. Notably, the SUN classification specifically highlights occlusive retinal vasculitis as a suggestive phenotype of OTB. We recently reported that that among the common ocular manifestations of uveitis associated with systemic TB, occlusive retinal vasculitis (15.7%) was the most frequent, followed by choroidal granuloma or tuberculoma (13.7%) [3].

Our understanding of the precise pathomechanism underlying retinal vasculitis, particularly tubercular retinal vasculitis (TRV), remains limited. In their initial efforts to classify uveitis, the SUN Working Group recognized retinal vasculitis as a term and clinical entity in need of further rigorous investigation [4]. They defined retinal vasculitis as “evidence of ocular inflammation and retinal vascular changes,” yet the exact nature of these vascular changes, and how to differentiate true inflammatory vasculitis from non-inflammatory vascular abnormalities, such as those seen in in diabetic retinopathy, has not been fully clarified. Retinal vasculitis can occur in a variety of contexts, including infectious uveitis and systemic diseases such as sarcoidosis or Behçet’s disease. Kaza *et al.* recently evaluated the clinical features of uveitis patients with visible *retinal vasculitis*, comparing those with evidence of active or latent tuberculosis (TB) to patients with non-TB-associated retinal vasculitis [5]. The study highlighted three features characteristic of TRV: subvascular lesions, focal vascular tortuosity, and occlusive characteristics [5]. However, further stratification based on treatment outcomes (with and without anti-tubercular treatment (ATT)) were not evaluated [5].

A study utilizing a zebrafish model elegantly demonstrated that systemic injection of mycobacteria can induce ocular inflammation resembling OTB, characterized by choroidal lesions and/or retinal vasculitis [6]. The study also observed that retinal vasculitis may occur even in the presence of an intact blood-retinal barrier (BRB). However, it remained unclear whether the mycobacteria crossed the BRB while contained within phagocytic cells or by another mechanism [6].

It can be speculated that the development of TRV may involve one or a combination of several pathomechanisms, as illustrated in Fig. 1. One potential mechanism is supported by our recent findings demonstrating that retinal endothelial cells (RECs) are permissive to *Mycobacterium tuberculosis (Mtb)* infection [7]. In patients with active TB, *Mtb* bacteremia has been estimated to occur in approximately 13.5% of adults, with an even higher prevalence of 15.5% among HIV-positive individuals [8]. This bacteremia may allow direct interaction between circulating *Mtb* and RECs. In our recent *in vitro* study *Mtb* infection of REC led to the activation of interferon signalling pathways [7], a response that resembles, at least in part, the innate immune activation observed in *Mtb*-infected retinal pigment epithelial (RPE) cells [7].

**Fig. 1:**
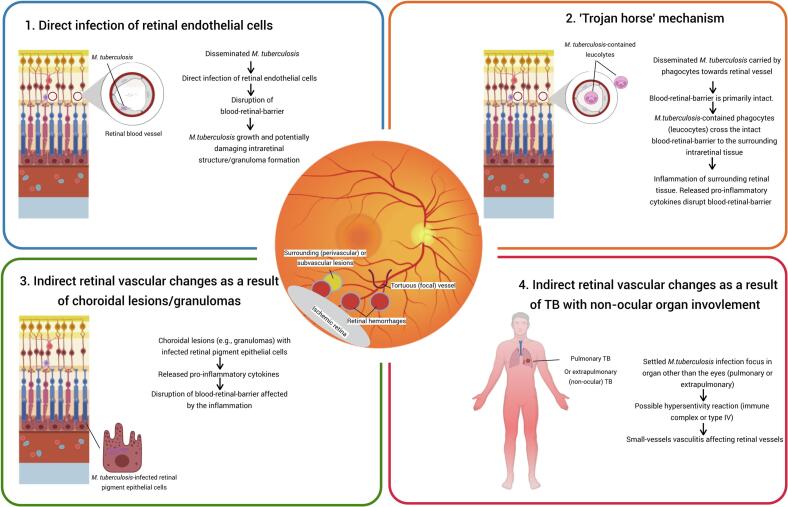
Hypothetical mechanisms involved in the development of tubercular retinal vasculitis. (Created with BioRender.com).

A second proposed mechanism underlying TRV is the “Trojan horse” pathway, wherein *Mtb*-infected phagocytes cross the BRB without initially disrupting its structural integrity. This mechanism, along with the direct infection of REC, has been implicated in the traversal of mycobacteria across the blood–brain barrier in zebrafish models, providing insight into the pathogenesis of TB meningitis and central nervous system (CNS) TB vasculitis [9]. Both direct REC infection and the “Trojan horse” pathway depend on the ESX-1 secretion system of the mycobacteria [9]. Importantly, the “Trojan horse” route may shield *Mtb* from direct interaction with RECs, while offering a protected intracellular niche within phagocytes that may favor bacterial survival [9]. Furthermore, histopathological studies of TRV by Basu et al. revealed the presence of intraretinal granulomas adjacent to vessel walls [10]. However, whether TRV primarily results from direct REC infection or from the “Trojan horse” route remains unclear.

An alternative mechanism to those described above is that TRV may arise secondary from choroidal lesions or infection of RPE cells lining the outer BRB, rather than involving the inner BRB. In this scenario, the clinically observed TRV could primarily reflect indirect inflammatory changes rather than direct infection of RECs or other intraretinal structures. A comparable concept has been proposed in vasculitis in birdshot retinochoroiditis, where retinal vascular changes are thought to be secondary to choroidal inflammation. The advent of indocyanine green angiography (ICGA) was pivotal in reshaping this understanding, revealing early hypofluorescent dark dots that were localized in the choroid, often before the characteristic birdshot lesions were visible with fundus examination [11]. Prior to the use of ICGA, retinal vasculitis was commonly noted ‘early’ in the disease course and was interpreted as a primary retinal manifestation. This shift in perspective might suggest that (a subset of) patients with TRV might similarly represent secondary retinal involvement driven by choroidal inflammation. Nevertheless, this mechanism may not explain all cases, as retinal vasculitis has also been observed to occur independently of choroidal inflammation [3].

A final proposed mechanism is that TRV may result from *Mtb* infection at a distant site. The association of small-vessel vasculitis with active TB elsewhere in the body has been suggested to represent a contributing factor in other vasculitic conditions, such as cutaneous leukocytoclastic vasculitis (CLV) [12]. Notably, vasculitic skin lesions in CLV have been reported to respond favorably to ATT [12]. Although the exact mechanism remains elusive, it is hypothesized this involves a hypersensitivity reaction to *Mtb* antigens [12].

In summary, TRV is a clinical entity that, in some patients, may represent a true manifestation of TB disease. This underscores the need for further investigation into its underlying mechanisms and the correlation with specific clinical features. Given the suboptimal diagnostic sensitivity of current molecular tests, such as polymerase chain reaction, for intraocular detection of *Mtb*, the development of alternative biomarkers, such as immune response-derived signatures indicative of active disease, must account for the distinct pathomechanisms that underlie TRV [13]. As research advances, a more refined understanding of OTB/TRV may help to identify which patients truly require ATT, even in the absence of direct microbiological identification of *Mtb* within ocular tissues, which remains a significant limitation in the current clinical practice.

## Funding sources

IP is supported by Indonesia Endowment Fund for Education (Lembaga Pengelola Dana Pendidikan–LPDP, no. 0004535/MED/D/19/lpdp2021). The funding source had no involvement in the collection, analysis, interpretation, writing of the report, or the decision to submit the article for publication.

## Declaration of generative AI in scientific writing

During the preparation of this work, the author(s) used ChatGPT (OpenAI, San Francisco, CA) for assistance in proofreading and editing the manuscript for grammar correction. None of the substantive content of this manuscript or its figure was generated through AI. The author(s) reviewed and edited the content as needed and take(s) full responsibility for the final manuscript.

## Ethical statement

Ethical approval is not necessary for the present perspective manuscript/study. No patient-related data or animal usage in the preparation of this manuscript.

## CRediT authorship contribution statement

**Ikhwanuliman Putera:** Conceptualization, Data curation, Formal analysis, Funding acquisition, Investigation, Methodology, Project administration, Resources, Visualization, Writing – original draft, Writing – review & editing. **Rina La Distia Nora:** Formal analysis, Investigation, Supervision, Validation, Writing – review & editing. **Saskia M. Rombach:** Formal analysis, Investigation, Supervision, Validation, Writing – review & editing. **P. Martin van Hagen:** . **Willem A. Dik:** Conceptualization, Formal analysis, Investigation, Methodology, Project administration, Resources, Supervision, Validation, Writing – review & editing.

## Declaration of competing interest

The authors declare that they have no known competing financial interests or personal relationships that could have appeared to influence the work reported in this paper.
